# No-tillage combined with deficit irrigation improves canopy photosynthesis and water use efficiency to stabilize yield in intercropped maize

**DOI:** 10.3389/fpls.2025.1712975

**Published:** 2026-01-27

**Authors:** Congcong Guo, Yan Wang, Xiaoyuan Bao, Hong Fan, Yali Sun, Wei He, Fuyang Cui, Chengxin Bai, Xinying Li, Cai Zhao

**Affiliations:** State Key Laboratory of Aridland Crop Science, College of Agronomy, Gansu Agricultural University, Lanzhou, China

**Keywords:** no-tillage, maize–pea intercropping, irrigation, canopy photosynthetic dynamics, water-use efficiency, source–sink dynamics

## Abstract

**Introduction:**

Water scarcity and uneven distribution of irrigation resources are major challenges for sustaining maize production in arid agro-ecosystems. While intercropping and conservation tillage have been individually recognized for enhancing crop productivity and resource efficiency, their integrated effects with irrigation management remain poorly understood. The long-term field platform was launched in 2015, and the trial was conducted in the northwest region in 2024, we embedded a three-factor split-plot experiment to evaluate the combined impacts of tillage (no-tillage, NT; conventional tillage, CT), planting pattern (maize–pea intercropping, IM; sole maize, SM), and irrigation regime (low, I1; medium, I2; high, I3) on maize yield, canopy photosynthetic dynamics, water-use efficiency, and photosynthetic enzyme gene expression.

**Methods:**

No-tillage intercropping under medium irrigation (NTIMI2) consistently achieved the highest yield, exceeding CTIMI2 and NTSMI2 by 10.5% and 27.2%, respectively, mainly through increases in ear number and thousand-kernel weight. Canopylevel analyses revealed that NTIMI2 sustained higher leaf area index, leaf area duration, crop growth rate, and net assimilation rate during silking–filling, thereby extending the photosynthetic functional period. These physiological advantages translated into greater assimilate supply and efficient partitioning, supported biochemically by the upregulation of nadp-mdh and nadp-me expression during grain filling. Importantly, NTIMI2 optimized the yield–water relationship: water-use efficiency was maximized and comparable yields were maintained relative to high irrigation, but with reduced water input.

**Discussion:**

Our findings provide mechanistic evidence that coordinated tillage and irrigation strategies regulate canopy source–sink dynamics and enzyme-mediated carbon assimilation, thereby reconciling the trade-off between yield stability and water conservation. This study highlights no-tillage intercropping with medium irrigation as a scalable pathway toward climate-resilient and water-efficient maize production in arid regions.

## 1 Introduction

Water scarcity has become one of the most critical constraints to sustainable agriculture worldwide, particularly in arid and semi-arid regions where crop production heavily depends on irrigation (Rosa et al., 2019). Climate change has intensified the frequency and severity of drought events, further aggravating the competition for limited water resources and threatening global food security (Leng and Hall, 2019; Lesk et al., 2016; Zhao et al., 2017). Maize (Zea mays L.), as a major cereal crop for food, feed, and industrial use, plays a pivotal role in ensuring food and nutritional security (Shiferaw et al., 2011). However, its high water requirement makes maize production especially vulnerable in water-limited environments. In northwestern China, such as the Hexi Corridor oasis irrigation zone, maize production relies predominantly on irrigation to achieve stable yields (Meier et al., 2021; Yuan et al., 2019). Yet the uneven spatial and temporal distribution of water resources, coupled with excessive groundwater exploitation, poses significant risks to the sustainability of this intensive production system. Addressing the dual challenge of maintaining high maize productivity while reducing water consumption has therefore become an urgent priority for regional and global agriculture.

Agronomic management practices such as tillage systems, planting patterns, and irrigation regimes are widely recognized as key levers for improving crop productivity and resource-use efficiency under water-limited conditions (Li et al., 2020; Liu et al., 2018). Conventional tillage often disrupts soil structure, accelerates moisture loss, and reduces soil organic matter, whereas conservation tillage practices such as no-tillage (NT) can enhance soil water retention, reduce evaporation, and mitigate drought stress, thereby sustaining photosynthetic activity during critical reproductive stages (Adil et al., 2024; Wang et al., 2020). In parallel, maize–legume intercropping has been demonstrated to improve canopy architecture, increase radiation interception, and promote resource complementarity (Brenner and Schmülling, 2015; Jaenisch et al., 2021; Zeng et al., 2022). The inclusion of legumes not only reduces interspecific competition but also enhances nitrogen availability through biological fixation, which collectively supports higher photosynthetic efficiency and grain yield (Duchene et al., 2017; Hu et al., 2018; Xu et al., 2020). Irrigation management further determines the extent to which these benefits are realized. While excessive irrigation may secure short-term yield gains, it often results in water wastage and ecological risks. In contrast, deficit or medium irrigation has been shown to maintain yield stability while significantly improving water-use efficiency (Almeida et al., 2013; Martínez-Goñi et al., 2023; Wang et al., 2020). Thus, integrating conservation tillage, intercropping, and optimized irrigation holds great promise for balancing crop productivity with sustainable water management in arid agro-ecosystems.

Despite the recognized benefits of conservation tillage, intercropping, and optimized irrigation when considered individually, their interactive effects on maize performance in arid regions remain insufficiently understood. Previous studies have primarily focused on single-factor comparisons, such as the influence of tillage on soil water dynamics, the effect of intercropping on canopy structure, or the impact of deficit irrigation on water-use efficiency (Glaze-Corcoran et al., 2020). However, little is known about how these practices interact to jointly regulate canopy photosynthetic traits, source–sink dynamics, and yield formation in maize–legume systems. In particular, there is a lack of integrative evidence linking field-level canopy processes (e.g., leaf area index, crop growth rate, net assimilation rate) with biochemical and molecular mechanisms (e.g., photosynthetic enzyme activities and gene expression) under different tillage–irrigation–cropping combinations. This knowledge gap constrains the development of mechanistic and scalable strategies to optimize maize production while minimizing water consumption in arid irrigated ecosystems.

To address these gaps, the present study systematically evaluated the combined effects of tillage practices, planting patterns, and irrigation regimes on maize production in an arid oasis irrigation zone of northwestern China. Specifically, we aimed to (i) quantify the impacts of no-tillage, intercropping, and deficit irrigation on maize grain yield and water-use efficiency; (ii) elucidate the dynamic responses of canopy photosynthetic traits—including leaf area index, leaf area duration, crop growth rate, and net assimilation rate—under different management combinations; and (iii) explore the physiological and molecular mechanisms underpinning yield stability by linking photosynthetic enzyme activities and gene expression with assimilate production and partitioning. By integrating field-scale and biochemical evidence, this study provides new mechanistic insights into how coordinated agronomic practices regulate source–sink relationships in intercropped maize. The findings are expected to inform the design of climate-resilient and water-efficient production strategies, contributing to sustainable intensification of maize systems in arid and semi-arid regions.

## 2 Materials and methods

### 2.1 Plant material

The maize cultivar used in the experiment was Xianyu 335 (*Zea mays* L.), and the pea cultivar was Longwan No. 1 (*Pisum sativum* L.). The chemical nitrogen fertilizers applied were urea and diammonium phosphate. White agricultural plastic film was used as mulch, with a width of 120 cm and a thickness of 0.01 mm.

### 2.2 Experiment design

This research is based on the long-term positioning experimental platform established in 2015. In 2024, field experiments were conducted in the northwest region. The experimental site was located at the Oasis Agricultural Research and Teaching Base of Gansu Agricultural University, located in Huangyang Town, Liangzhou District, Wuwei City, Gansu Province (37°30′N, 103°5′E). The site lies in a cold temperate arid climatic zone, with an average annual precipitation of approximately 156 mm, annual evaporation of about 2400 mm, annual sunshine duration of 2969.2 hours (h), mean air temperature of 7.2°C, and a frost-free period of 156 days. The region is rich in solar radiation, making it suitable for intercropped maize cultivation. Local agricultural production is dominated by conventional tillage with plowing, and plastic film mulching is widely adopted.

The soil type at the experimental site was irrigated desert soil. In 2024, the nutrient contents of the plow layer were as follows: total nitrogen 0.89 g·kg^-^¹, available phosphorus 24.98 mg·kg^-^¹, available potassium 138.44 mg·kg^-^¹, organic matter 14.53 g·kg^-^¹, and bulk density 1.24 g·cm^-^³. Variations in temperature and precipitation during the entire growing season in 2024 are shown in **Supplementary Figure S1**.

Based on a long-term experiment initiated in 2015, this study was conducted using a split–split plot design with three factors. The main plots included two tillage practices: no-tillage (NT: direct seeding on the residual film after maize harvest without tillage) and conventional tillage (CT: deep plowing after maize harvest followed by land preparation and new film mulching before sowing in the following year). The subplots consisted of two cropping patterns: intercropped maize (IM) and monocropped maize (SM). The sub-subplots included three irrigation levels: low irrigation (I1, 4500 m³·hm^-^²), medium irrigation (I2, 4950 m³·hm^-^²), and high irrigation (I3, 5400 m³·hm^-^²), with the high irrigation level corresponding to the local conventional practice. In total, 12 treatments were established with three replicates, resulting in 36 plots. Each plot had an area of 63 m² (7 m × 9 m). Treatment codes are provided in **Supplementary Table S1**.

The planting density of monocropped maize was 90,000 plants·hm^-^², with a row spacing of 40 cm and a plant spacing of 27 cm. In the maize–pea intercropping system, a 3:4 planting ratio (three rows of maize to four rows of pea) was adopted, with a row spacing of 25 cm between maize and pea. The planting density of intercropped maize was 52,000 plants·hm^-^², while that of pea was 760,000 plants·hm^-^², with an inter-row ratio of 8:11 between maize and pea strips. Sowing was carried out on April 18, 2024, for maize and April 7, 2024, for pea, with harvesting on September 27 and July 6, 2024, respectively. After harvest, all crop residues were removed from the field.

All irrigation treatments were carried out using drip irrigation under plastic film, with irrigation volumes monitored by water meters. Specific irrigation quotas at different growth stages are provided in **Supplementary Table S2**. The maize fertilization regime followed local conventional management, with a total nitrogen application rate of 360 kg·hm^-^², split among the basal stage, the big trumpet stage, and the grain-filling stage at a ratio of 3:5:2. Phosphorus fertilizer was applied at an N\:P ratio of 2:1, corresponding to 180 kg·hm^-^², and was incorporated entirely as a basal application. For pea, the total nitrogen and phosphorus application rates were 90 kg·hm^-^² and 45 kg·hm^-^², respectively, with all fertilizers applied as a basal dose at sowing. Given the high potassium content in the local soil, no potassium fertilizer was applied. Pest, disease, and weed management were performed according to local conventional practices.

### 2.3 Yield and biomass

At maturity, grain yield was determined by manually harvesting a 5 m segment along the row direction from the central rows of each plot. To minimize border effects, only continuous stands without missing plants were selected. The sampling belt consisted of multiple rows within a fixed width of 1.4 m, covering the actual planting configuration. This resulted in a harvested area of 7 m² (5 m × 1.4 m). All ears within the sampling area were hand-harvested and immediately threshed in the field. The fresh grain weight, after removing impurities, was recorded using an electronic balance with a precision of 0.01 kg.

Note: All ‘row spacing’ values (40 cm or 25 cm) describe the planting configuration; the 1.4 m dimension used in yield sampling is the fixed belt width within which all consecutive rows were harvested.

Approximately 500 g of fresh kernels were randomly collected from each plot to determine grain moisture content. Samples were first oven-dried at 105 °C for 30 min to inactivate enzyme activity, and then further dried at 80 °C to a constant weight. Kernel dry weight was recorded using an electronic balance with a precision of 0.01 kg. According to the *National Standard for Maize Yield Determination* (GB/T 22022-2008, China), the actual moisture content was calculated, and grain yield was adjusted to the standard level of 14% moisture. Final yield was expressed in kilograms per hectare (kg·hm^-^²).

### 2.4 Water use efficiency

The evapotranspiration (ET) was calculated as follows (Equation 1):

(1) Evapotranspiration=Precipitation+amount of irrigation water applied

Water use efficiency was calculated as follows (Equation 2):

(2) $$\mathrm{Water use efficiency}=\frac{\mathrm{Grain yield}\left(\text{kg}\cdot {\text{hm}}^{-2}\right)}{\text{Evapotranspiration}}$$

### 2.5 Canopy photosynthetic physiology

At the seedling, jointing, trumpet, silking, grain-filling, milk, and maturity stages of maize, the leaf area index, photosynthetic potential, crop growth rate, and net assimilation rate were measured. In each plot, three representative plants were randomly selected from the inner rows to avoid border effects. All leaf samples were collected between 09:00 and 11:00 on clear, calm days; sampling was postponed at least 24 h after any rainfall or irrigation to minimise diurnal and moisture-driven variability.

Leaf area index: Individual leaves were detached, and their areas were measured using a portable leaf area meter (e.g., LI-3100C, LI-COR Inc., Lincoln, NE, USA). The total leaf area per plant was calculated as the sum of all leaf areas.

Leaf area duration was calculated as follows (Equation 3):

(3) $$\text{Leaft area duration}=\sum_{\text{i}=1}^{n}{\text{(LAI}}_{i}\times {\text{D}}_{i})$$

Where, represents the mean leaf area index at the i-th growth stage, and denotes the number of days during the i-th growth stage.

Crop growth rate (kg·hm-2 d-1) was calculated to describe the increase in aboveground dry matter per unit land area and per unit time. It was determined between two consecutive sampling dates using the following formula (Equation 4):

(4) $$\text{Cropt growth rate}=\frac{{\text{W}}_{2}-{\text{W}}_{1}}{{\text{t}}_{2}-{\text{t}}_{1}}$$

Where and ${\text{ W}}_{2}$ are the aboveground dry matter weights at times and ${\text{ t}}_{2}$ respectively.

Net assimilation rate (g ·m^-2^ d-1) describes the rate of dry matter accumulation per unit leaf area per unit time. It was calculated according to the classical growth analysis method as follows (Equation 5):

(5) $$\text{Net assimilation rate}=\frac{{\text{lnL}}_{2}-{\text{lnL}}_{1}}{{\text{LAI}}_{2}-{\text{LAI}}_{1}}\times \frac{{\text{W}}_{2}-{\text{W}}_{1}}{{\text{t}}_{2}-{\text{t}}_{1}}$$

where ${\text{W}}_{1}$ and ${\text{ W}}_{2}$ are the aboveground dry matter weights at times and ${\text{ t}}_{2}$ respectively, and and are the corresponding leaf areas.

### 2.6 Relative expression of key photosynthetic enzyme genes

To analyze the expression dynamics of key photosynthetic enzymes in maize leaves under different tillage practices, cropping patterns, and irrigation regimes, samples were collected at three growth stages: jointing, silking, and grain filling. For each treatment, three maize plants with uniform growth were randomly selected. All leaf samples were collected between 09:00 and 11:00 on clear, calm days; sampling was postponed at least 24 h after any rainfall or irrigation to minimise diurnal and moisture-driven variability. At the jointing stage, the second fully expanded leaf from the top was sampled, while at the silking and grain-filling stages, the middle section of the ear leaf was collected. After removing the veins, 0.2 g of fresh tissue was rapidly weighed, frozen in liquid nitrogen, and stored at −80°C.

Total RNA was extracted from maize leaf samples using the FastPure^®^ Plant Total RNA Isolation Kit (Nanjing, China). RNA quality was assessed using an Agilent 2100 Bioanalyzer and verified by RNase-free agarose gel electrophoresis. Total RNA was reverse-transcribed into cDNA using the HiScript^®^ II Q RT SuperMix for qPCR (+gDNA wiper) kit (Nanjing, China). The cDNA synthesis reaction mixture (20 µL) contained 4 µL of 5× HiScript II qRT SuperMix II, 4 µL of 4× gDNA Wiper Mix, 12 µL of RNase-free H_2_O, and 1000 ng of total RNA. The reaction conditions were 50°C for 15 min and 85 °C for 5 s. The resulting cDNA was stored at −20°C until further use.

Subsequently, quantitative real-time PCR (qRT-PCR) was performed. Maize coding sequences were obtained from the Maize Genetics and Genomics Database (http://www.maizegdb.org/). Primers were designed using Primer Premier 5.0 (Canada) and synthesized by Shanghai OE Biotech Co., Ltd. (primer sequences are listed in **Table 1**). The reactions were conducted using a Taq Pro Universal SYBR qPCR Master Mix kit on a LightCycler^®^ 480 II real-time PCR system (Roche, Switzerland). The reaction mixture (20 µL) consisted of: 10 µL 2× Taq Pro Universal SYBR qPCR Master Mix, 0.4 µL 10 µM Primer 1, 0.4 µL 10 µM Primer 2, 1 µL cDNA, and 8.5 µL nuclease-free H_2_O. The PCR program was as follows: 95 °C for 30 s; followed by 40 cycles of 95°C for 5 s and 60 °C for 30 s. Relative gene expression levels were calculated using the 2^-^(ΔΔCt) method, with the maize *ZmActin* gene serving as the internal control. Each treatment included three biological replicates. Detailed primer sequences are provided in **Supplementary Table S3**.

**Table 1:** Three factors of maize yield under different treatments.

Tillageinitiatives	Plantingpattern	Irrigationamounts	Earnumbers(earm^-2^)	Kernelnumberperspilk	1000-kernelweight(g)
NT	IM	I1	8.1±0.12 d	562.0±18.6 c	436.0±7.5 d
		I2	9.5±0.21 a	643.5±21.2 ab	489.1±18.6 a
		I3	9.6±0.06 a	652.2±17.0 a	194.6±5.0 a
	SM	I1	7.4±0.15 e	528.3±6.9 d	410.7±13.2 f
		I2	9.0±0.10 bc	586.9±14.9 c	461.9±16.5 bc
		I3	9.1±0.20 b	619.7±19.3 b	463.0±21.6 b
CT	IM	I1	7.5±0.15 e	523.5±24.4 d	413.9±8.6 ef
		I2	8.8±0.12 c	620.8±33.0 b	460.2±12.7 bc
		I3	9.1±0.17 b	631.5±21.6 ab	465.5±18.8 b
	SM	I1	7.0±0.10 f	492.1±20.7 e	388.5±7.0 g
		I2	8.3±0.26 d	583.4±16.4 c	343.4±13.5 de
		I3	9.0±0.10 bc	617.9±9.0 b	440.6±10.0 cd
Variance analysis					
Tillage initiatives (T)			**	NS	**
Planting pattern (P)			**	NS	*
Irrigation amounts (I)			**	*	*
T×P			*	NS	**
T×I			*	NS	*
P×I				NS	*
T×P×I			NS	NS	NS

NT and CT represent no-tillage and conventional tillage, respectively. IM and SM were intercrop and monocrop maize, respectively. I1, I2, and I3 represent low, medium, and high irrigation amount, respectively. Differences between treatments are denoted by different lowercase letters, with ** and * indicating significant effects of the experimental factors on the parameter at the 0.01 and 0.05 significance levels, respectively, while NS denotes no significant effect of the experimental factors on the parameter.

### 2.7 Statistics and analysis

Data were recorded and organized using Microsoft Excel 2019. Duncan’s multiple range tests were performed in SPSS 21.0. Figures and correlation analyses were generated with Origin 2019b and GraphPad Prism 9.0. Data are expressed as mean ± standard error (mean ± SE). One-way ANOVA and the least significant difference (LSD) test were used to determine statistical significance at the 95% or 99% confidence level. Figure assembly was done in Adobe Illustrator 2020.

Results

### 3.1 Effects of no-tillage and deficit irrigation on grain yield and yield components of intercropped maize

**Figure 1:**
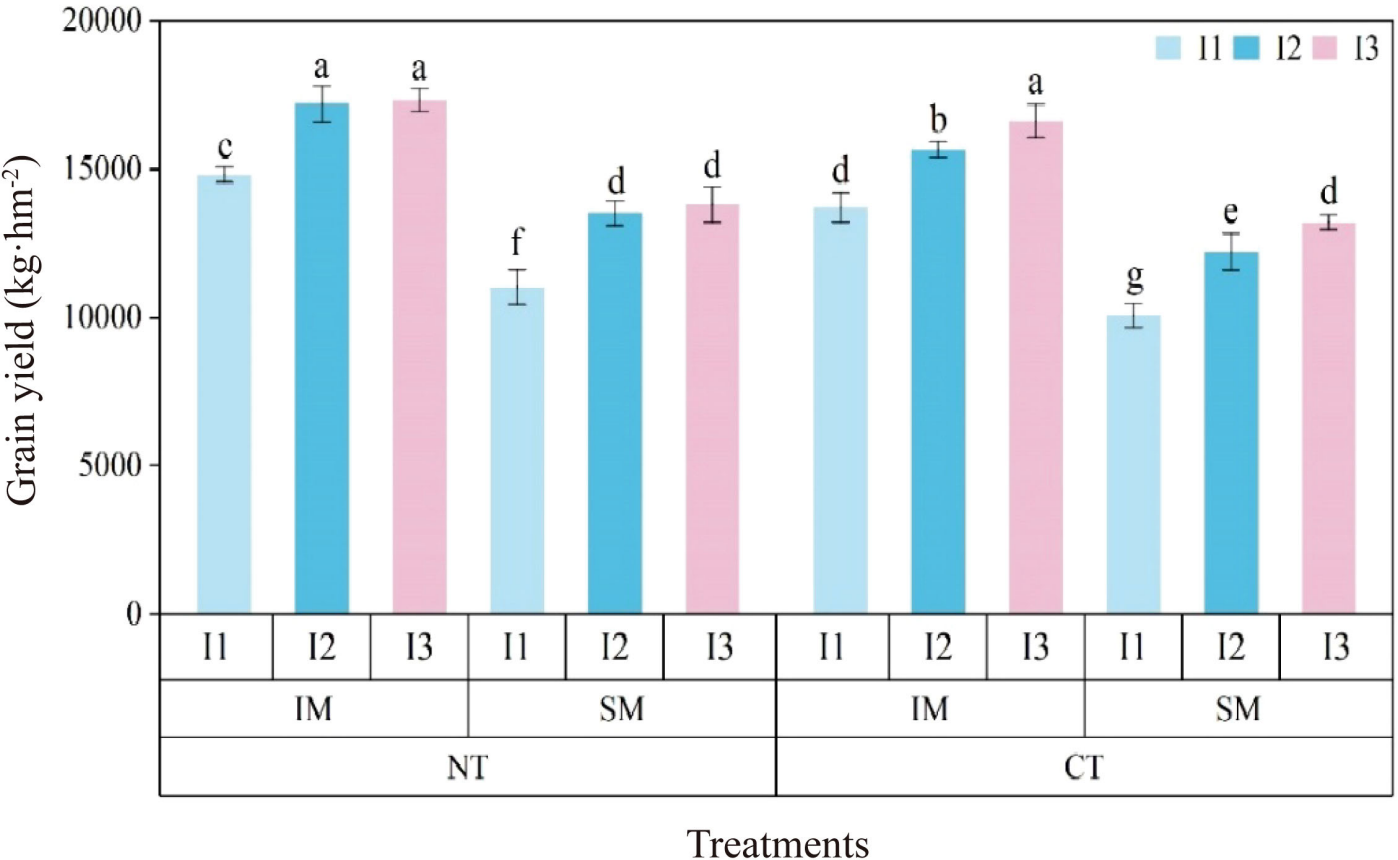
Effects of no-tillage and deficit irrigation on grain yield of intercropped maize. NT and CT represent no-tillage and conventional tillage, respectively. IM and SM were intercrop and monocrop maize, respectively. I1, I2, and I3 represent low, medium, and high irrigation amount, respectively. Differences between treatments are denoted by different lowercase letters, with a significance level of 0.05, and the same applies hereafter.

NT increased grain yield by 7.8% compared with CT, and IM enhanced yield by 29.5% relative to SM. Yield decreased with reduced irrigation, with I1 reducing yield by 18.9% compared with I3, whereas the difference between I2 and I3 was not significant. Among treatment combinations, NTIMI2 produced 10.5% and 27.2% higher yields than CTIMI2 and NTSMI2, respectively; NTIMI1 yielded 14.5% less than NTIMI3, while NTIMI2 and NTIMI3 showed no significant difference (**Figure 1**).

The yield components exhibited trends consistent with those of grain yield (**Table 1**). Tillage practice, cropping pattern, and irrigation level all had significant effects on ear number and 1,000-kernel weight, but not on kernels per ear. Compared with CT, NT increased ear number per unit area by 6.0%; compared with SM, IM increased ear number by 5.8%. Ear number under I1 was 11.5% lower than under I3, while there was no significant difference between I2 and I3. Interaction analysis showed that the ear number of NTIMI2 was 8.0% and 5.2% higher than those of CTIMI2 and NTSMI2, respectively.

For 1,000-kernel weight, NT significantly increased values by 5.8% compared with CT, while IM increased values by 6.2% compared with SM. Kernel weight decreased with reduced irrigation, with I1 being 11.5% lower than I3, whereas I2 did not differ significantly from I3. Among the treatment combinations, NTIMI3 and NTIMI2 increased 1,000-kernel weight by approximately 6% compared with their corresponding CT treatments, and by 6.8% and 5.9%, respectively, compared with monocropping. However, NTIMI1 showed a significant 11.8% reduction compared with NTIMI3, while NTIMI2 and NTIMI3 were not significantly different.

### 3.2 Effects of no-tillage and deficit irrigation on biomass of intercropped maize

**Figure 2:**
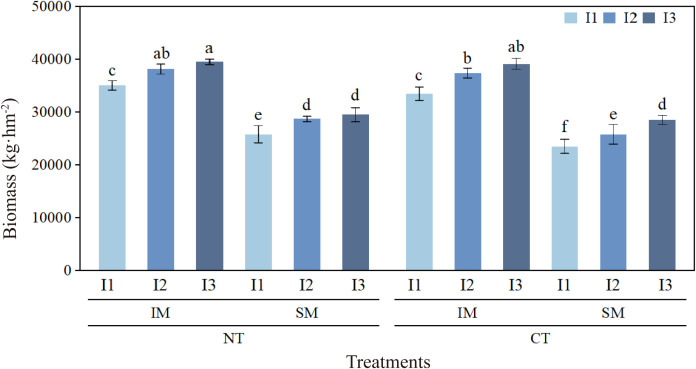
Effects of no-tillage and deficit irrigation on biomass of intercropped maize. See **Figure 1** for treatment codes. Differences between treatments are denoted by different lowercase letters, with a significance level of 0.05, and the same applies hereafter.

Under the same land area, intercropping increased biomass by 37.8% compared with monocropping, while NT increased biomass by 5.4% compared with CT. Biomass under I1 and I2 decreased by 4.0% and 5.1%, respectively, compared with high irrigation (I3). Treatment comparisons showed that NTIMI2 did not differ significantly from CTIMI2 but was slightly higher overall; NTIMI1 increased by 4.7% compared with CTIMI1. Under the same land area, NTIMI2 and NTIMI1 produced significantly higher biomass than NTSMI2 and NTSMI1 by 32.8% and 35.8%, respectively. However, NTIMI1 was 8.1% and 11.3% lower than NTIMI2 and NTIMI3, respectively, while NTIMI2 and NTIMI3 showed no significant difference (**Figure 2**).

### 3.3 Effects of no-tillage and deficit irrigation on water use efficiency of intercropped maize

**Figure 3:**
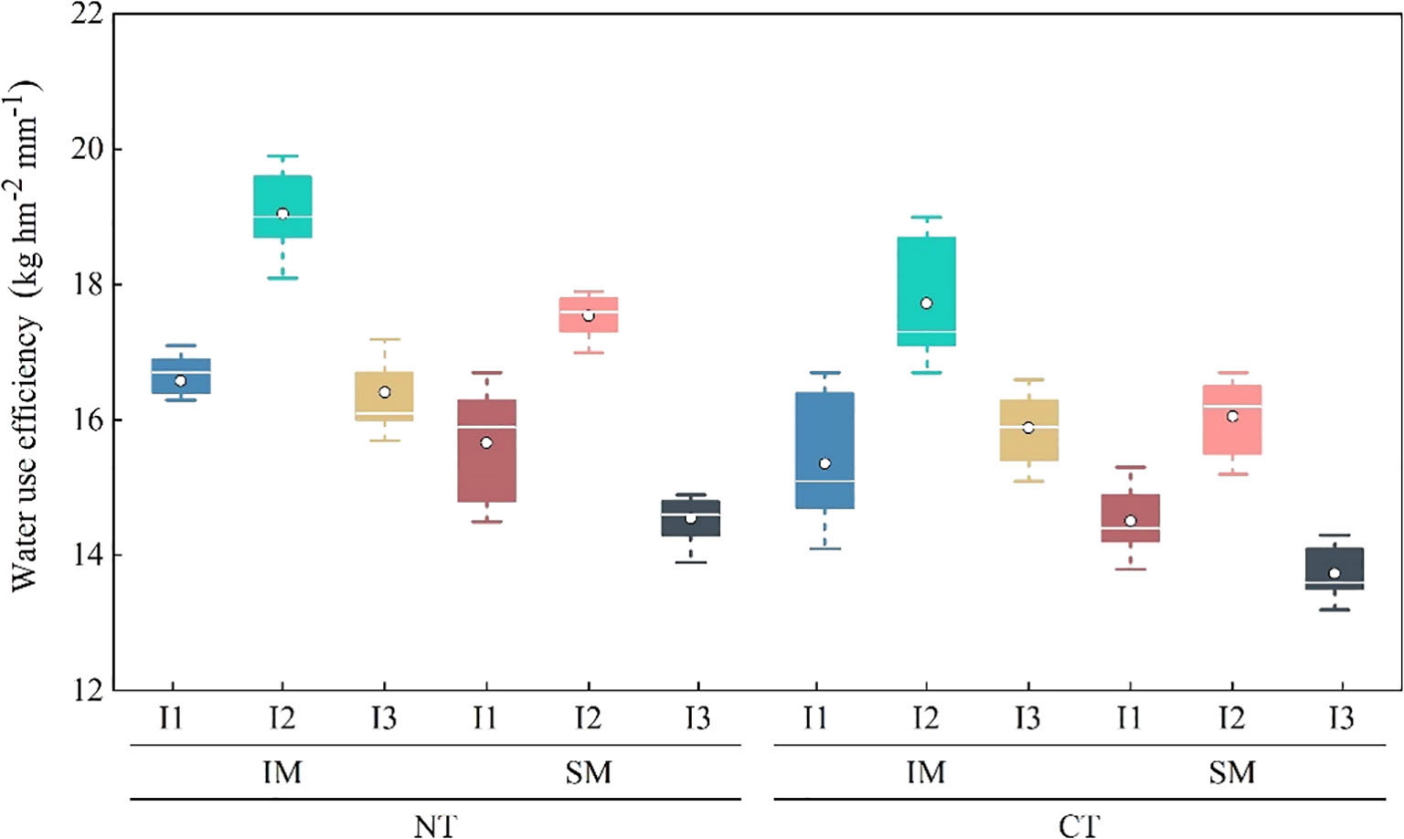
Effects of no-tillage and deficit irrigation on water use efficiency of intercropped maize. See **Figure 1** for treatment codes. Differences between treatments are denoted by different lowercase letters, with a significance level of 0.05, and the same applies hereafter.

NT increased water use efficiency by 6.8% compared with CT, and IM improved water use efficiency by 9.9% compared with SM. Among irrigation treatments, I2 performed best, increasing water use efficiency by 16.4% and 11.7% compared with I3 and I1 irrigation, respectively. For specific treatment combinations, NTIMI2 improved water use efficiency by 7.2% and 8.3% compared with CTIMI2 and NTSMI2, respectively, while NTIMI1 increased water use efficiency by 8.0% and 6.1% compared with CTIMI1 and NTSMI1. Within NT intercropping treatments, NTIMI2 showed significantly higher water use efficiency than NTIMI3 and NTIMI1, with increases of 16.0% and 12.7%, respectively (**Figure 3**).

### 3.4 Effects of no-tillage and deficit irrigation on leaf area index of intercropped maize

**Figure 4:**
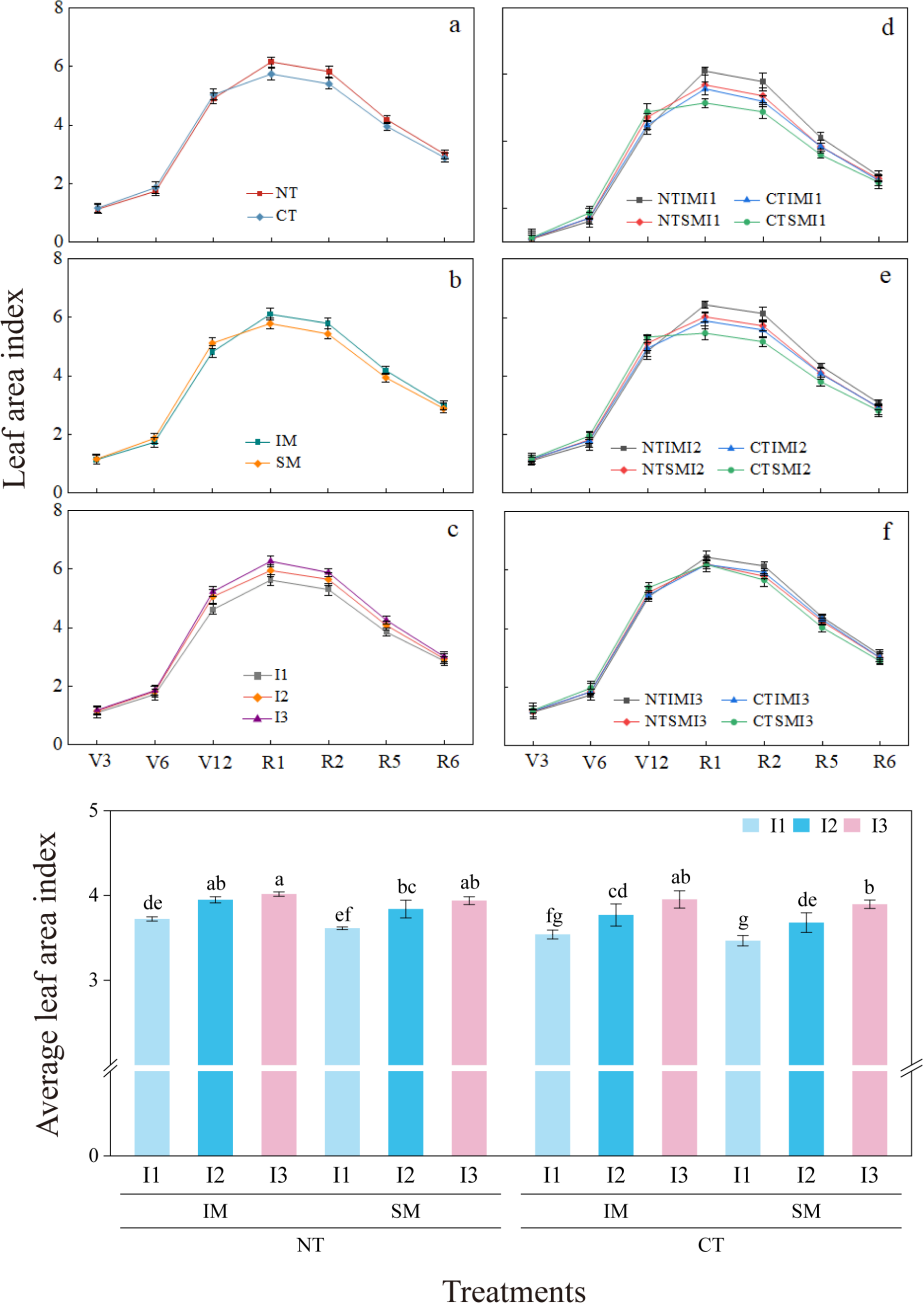
**(a–f)** Effects of no-tillage and deficit irrigation on dynamic leaf area index and average leaf area index of intercropped maize. Differences between treatments are denoted by different lowercase letters, with a significance level of 0.05, and the same applies hereafter. Note: See **Figure 1** for treatment codes. V3, V6, V12, R1, R2, R5, R6 represent seeding, jointing, big flare, silking, filling, doughing, and maturing stages, respectively.

In terms of developmental dynamics, leaf area index under NT was lower than CT at the jointing stage, but significantly higher (by 5.9%–8.2%) from silking to milk stages. Similarly, leaf area index under IM was lower than SM from jointing to trumpet stages, but increased by 6.1%–6.4% from silking to milk stages. Among irrigation treatments, I1 reduced leaf area index by 7.2%–11.7% compared with I3 from jointing to milk stages, while I2 did not differ significantly from I3. Treatment comparisons showed that from silking to milk stages, NTIMI2 exhibited 6.5%–10.0% and 5.8%–7.3% higher leaf area index than CTIMI2 and NTSMI2, respectively; NTSMI2 was 7.9%–10.4% higher than CTSMI2. However, NTIMI1 showed a 5.4%–6.7% reduction compared with NTIMI3, while NTIMI2 and NTIMI3 did not differ significantly. In terms of mean leaf area index, NTIMI2 and NTIMI3 were comparable, whereas CTIMI2 was 4.6% lower than CTIMI3, and NTIMI2 was 4.8% higher than CTIMI2 (**Figure 4**).

### 3.5 Effects of no-tillage and deficit irrigation on leaf area duration of intercropped maize

**Figure 5:**
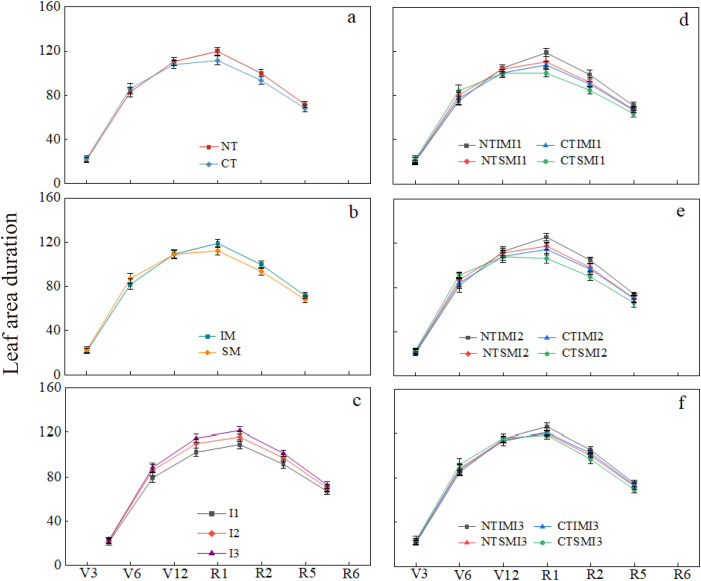
**(a–f)** Effects of no-tillage and deficit irrigation on leaf area duration of intercropped maize. See **Figures 1** and **4** for treatment codes.

Compared with CT, NT reduced leaf area duration by about 5.1% from the seedling to jointing stage, but increased it by 7.1%–7.6% from silking to milk stages. Under the same tillage and irrigation conditions, IM showed lower leaf area duration than SM from jointing to trumpet stages, but was 6.1%–6.5% higher from silking to milk stages. With the same tillage and cropping pattern, I1 reduced leaf area duration by 7.2%–10.9% compared with I3 from jointing to milk stages, while no significant difference was observed between I2 and I3. Treatment comparisons indicated that NTIMI2 was 5.5% lower than CTIMI2 at the seedling to jointing stage, but significantly higher by 8.5%–9.6% from silking to milk stages. Compared with NTSMI2, NTIMI2 was lower at the early stage, but higher by 6.7%–7.0% from silking to milk stages. NTIMI1 decreased by 12.7%–15.5% compared with NTIMI3 during silking to milk stages, while NTIMI2 and NTIMI3 showed no significant difference (**Figure 5**).

### 3.6 Effects of no-tillage and deficit irrigation on crop growth rate of intercropped maize

**Figure 6:**
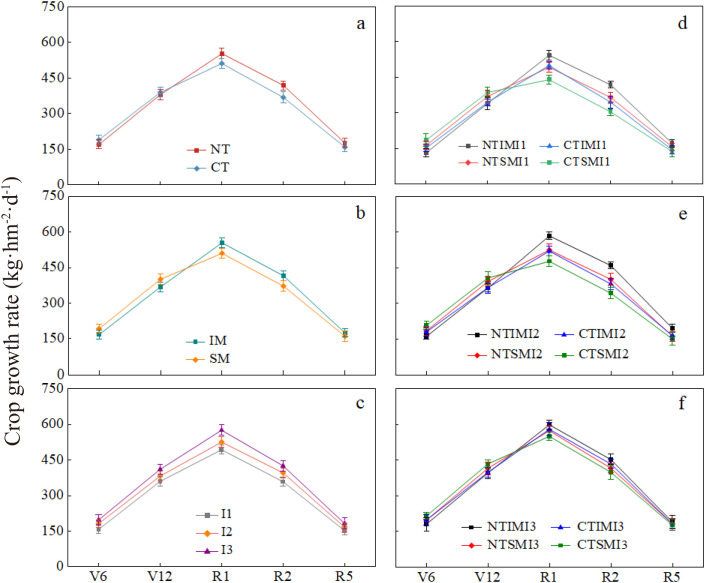
**(a–f)** Effects of no-tillage and deficit irrigation on group growth rate of intercropped maize. See **Figures 1** and **4** for treatment codes.

Under the same cropping pattern and irrigation level, crop growth rate under NT was 10.6% lower than CT at the jointing stage, but increased by 8.4%–14.5% from silking to milk stages. Similarly, IM showed 9.5%–10.0% lower crop growth rate than SM from jointing to trumpet stages, but was 8.1%–13.2% higher from silking to milk stages. For irrigation treatments, crop growth rate under I1 and I2 was reduced by 12.4%–20.3% and 7.1%–8.7%, respectively, compared with I3 during silking to milk stages. In terms of treatment combinations, NTIMI2 was 10.3% lower than CTIMI2 at the jointing stage, but increased by 12.4%–20.4% from silking to milk stages. Compared with NTSMI2, NTIMI2 was lower at the early stage, but 11.4%–21.3% higher during silking to milk stages. NTIMI1 showed a 7.6%–9.5% reduction compared with NTIMI3, while NTIMI2 and NTIMI3 did not differ significantly (**Figure 6**).

### 3.7 Effects of no-tillage and deficit irrigation on net assimilation rate of intercropped maize

**Figure 7:**
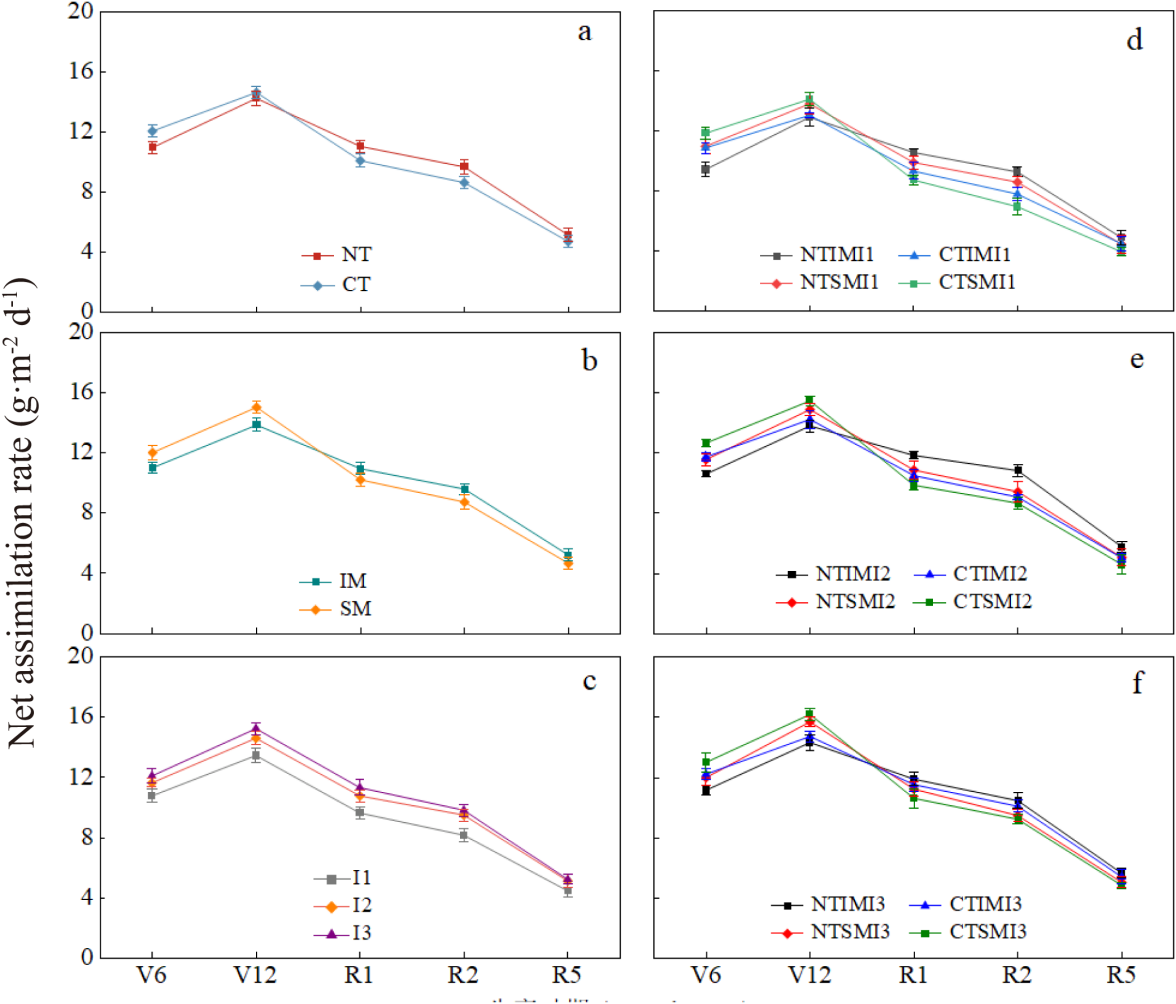
**(a–f)** Effects of no-tillage and deficit irrigation on net assimilation rate of intercropped maize. See **Figures 1** and **4** for treatment codes.

Under the same cropping pattern and irrigation level, NT showed 9.2% lower net assimilation rate than CT at the jointing stage but increased by 9.1%–12.8% from silking to milk stages. With the same tillage and irrigation conditions, intercropping exhibited lower net assimilation rate than monocropping before silking, but was 7.2%–11.7% higher from silking to milk stages. As irrigation decreased, I1 reduced net assimilation rate by 11.1%–17.0% compared with I3 from jointing to milk stages, while no significant difference was observed between I2 and I3. Interaction effects indicated that NTIMI2 was lower than CTIMI2 at the jointing stage, but increased by 12.7%–19.5% from silking to milk stages. Compared with NTSMI2, NTIMI2 was 8.9%–14.8% higher during silking to milk stages. NTIMI1 decreased by 11.2%–13.0% compared with NTIMI3 in the same period, while NTIMI2 and NTIMI3 showed no significant difference. Meanwhile, CTIMI2 was 6.8%–10.2% lower than CTIMI3 (**Figure 7**).

### 3.8 Effects of no-tillage and deficit irrigation on the relative expression of key photosynthetic enzyme genes in intercropped maize

**Figure 8:**
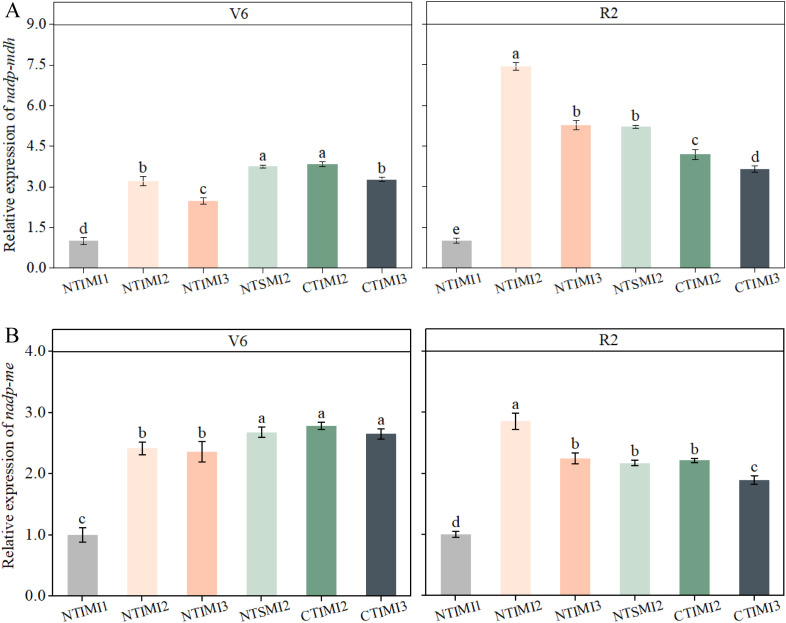
**(A, B)** Effects of no-tillage and deficit irrigation on relative expression of nadp-mdh and nadp-me genes in intercropped maize leaves. See **Figure 1** for treatment codes. Differences between treatments are denoted by different lowercase letters, with a significance level of 0.05, and the same applies hereafter. Treatments are the same as those given in **Figure 4**.

At the jointing stage, NTIMI2 showed a downregulation of approximately 13%–16% compared with CTIMI2 and NTSMI2. In contrast, a significant upregulation was observed at the grain-filling stage, where NTIMI2 increased by 29.1%–77.8% relative to CTIMI2 and by 31.9%–42.8% relative to NTSMI2. Across irrigation levels, no significant difference was detected between NTIMI2 and NTIMI3 at the jointing stage, whereas at the grain-filling stage NTIMI2 was significantly higher than NTIMI3, with increases of 27.0%–41.0%. CT treatments exhibited a similar trend (**Figure 8**).

### 3.9 Correlation between grain yield and irrigation amount under no-tillage intercropping

**Figure 9:**
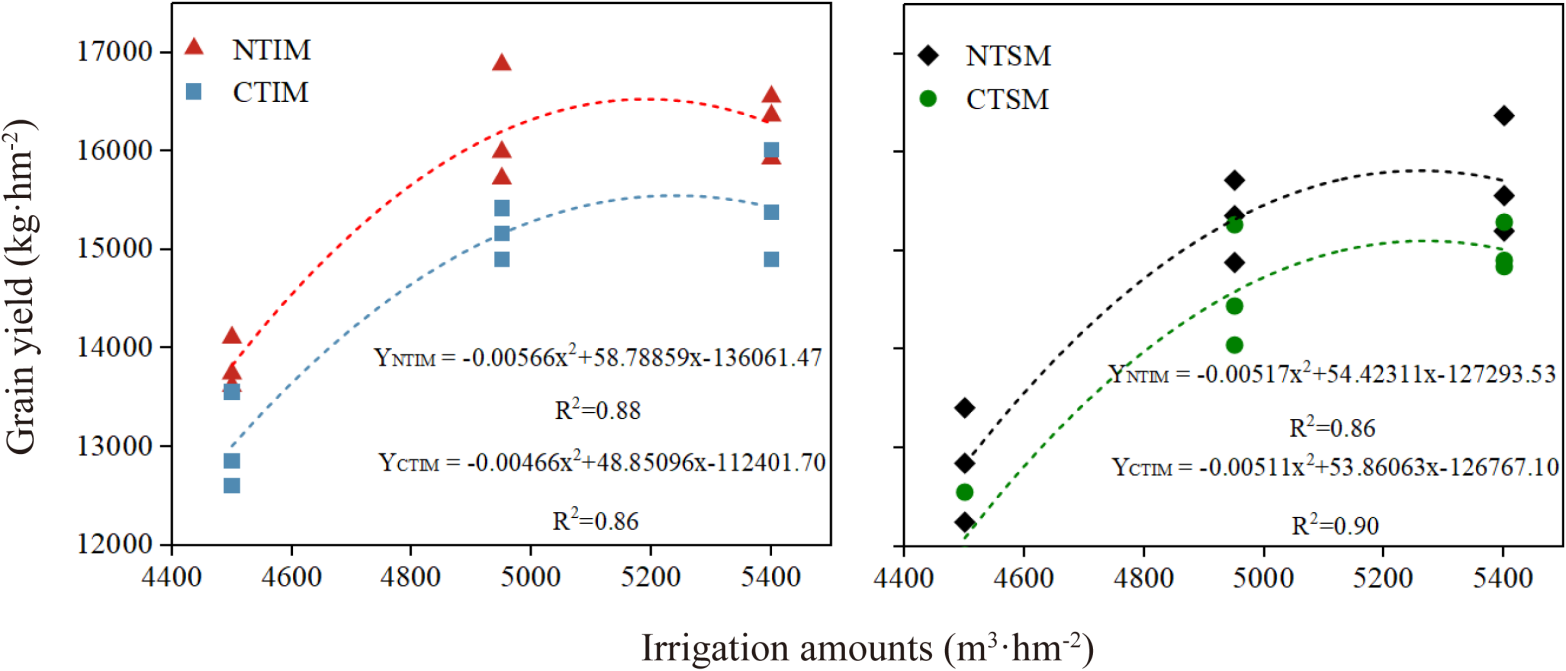
Regression analysis of grain yield and irrigation amount for intercropped maize under no-tillage and deficit water.

Regression analysis showed that maize grain yield exhibited a parabolic increase with irrigation amount under different combinations of tillage and cropping patterns, with high fitting accuracy across all groups (R² > 0.86; **Figure 9**). Under NTIM, the optimal irrigation amount was 5193 m³·hm^-^², corresponding to a yield of 16,593.0 kg·hm^-^². For CTIM, NTSM, and CTSM, the optimal irrigation amounts were 5242, 5263, and 5270 m³·hm^-^², with corresponding yields of 15,624.9, 15,929.6, and 15,158.9 kg·hm^-^², respectively. Compared with other treatments, NTIM increased the optimal yield by 6.1%, 4.2%, and 9.5%, respectively, while requiring the lowest irrigation amount, close to the medium irrigation level. These findings indicate that NT combined with intercropping under medium irrigation can achieve high water use efficiency and grain yield, representing the optimal model for stable and increased maize production in arid regions.

## 4 Discussion

### 4.1 Yield formation under no-tillage and deficit irrigation

Grain yield stability is the primary target of maize-based intercropping systems under limited water supply. In the present study, no-tillage combined with intercropping significantly increased maize yield compared with conventional tillage, particularly under medium irrigation (NTIMI2), where grain yield was 10.5% and 27.2% higher than CTIMI2 and NTSMI2, respectively. These findings are consistent with previous reports that conservation tillage enhances soil structure and water retention, thereby sustaining reproductive growth and yield formation under water-limited conditions (Li et al., 2025; Q. Zhang et al., 2022). The higher ear number and thousand-kernel weight observed in NTIMI2 indicate that no-tillage intercropping not only increased sink capacity but also improved assimilate supply during grain filling. This result corroborates earlier studies suggesting that optimized tillage and irrigation practices alleviate the decline in chlorophyll content and maintain photosynthetic activity during the critical silking and filling stages (Li et al., 2024; Wang et al., 2022).

Interestingly, although low irrigation (I1) markedly reduced yield, medium irrigation (I2) maintained comparable productivity to high irrigation (I3), highlighting that excessive water input is not necessary for stable yield in oasis agro-ecosystems. Similar parabolic responses of maize yield to irrigation amount have been reported in arid regions, where the optimum irrigation threshold was close to medium irrigation levels (Martínez-Goñi et al., 2023; Niu et al., 2023). Taken together, our results suggest that no-tillage intercropping, when integrated with medium irrigation, is an effective strategy to secure high maize productivity while minimizing water inputs in arid irrigated regions.

### 4.2 Regulation of canopy photosynthetic traits of intercropped maize under no-tillage and deficit irrigation

Improving canopy structure and optimizing population photosynthetic performance are key approaches to enhancing crop photosynthetic efficiency and biomass production capacity (Lai et al., 2025). In this study, the integrated management of no-tillage and intercropping effectively mitigated the decline in maize canopy photosynthetic productivity under limited irrigation, while reasonably regulating photosynthetic physiological traits during the coexistence period and after pea harvest. This significantly increased leaf area index, leaf area duration, and net assimilation rate of maize from silking to grain filling. Since leaf area index, leaf area duration, and net assimilation rate are critical indicators of the photosynthetic source, and their magnitude is directly associated with grain yield, expanding the photosynthetic source in the early growth stages and maintaining a larger green leaf area in the later stages constitute an essential foundation for yield improvement (Straube, 2023; Zhang et al., 2020).

At the population level, a key factor for intercropping systems to achieve stable and increased crop yield is the maintenance of relatively high photosynthetic levels during the late growth stages (Straube, 2023). The results of this study showed that maize leaf area index, leaf area duration, and net assimilation rate from silking to grain filling were significantly higher under intercropping than monocropping, which is consistent with previous findings. Intercropped maize exhibited significantly greater leaf area index, leaf area duration, and net assimilation rate after silking compared with monocropped maize, thereby better coordinating changes in photosynthetic source supply during growth and extending its functional duration (Chen et al., 2024). The underlying reasons may be as follows: First, in the maize–pea intercropping system, strong complementarity between the two crops and efficient utilization of light and heat resources enabled full exploitation of spatial niches at different canopy layers. This not only avoided excessive competition for water and nutrients but also promoted leaf expansion, thereby enhancing canopy photosynthetic efficiency, leaf area index, leaf area duration, and net assimilation rate (Chen et al., 2024). Second, intercropping improves soil fertility. In cereal–legume intercropping systems, legumes can fix atmospheric nitrogen and provide an additional nitrogen source for maize, which promotes growth and development of the cereal crop, supports leaf expansion, and ultimately improves leaf area index, leaf area duration, and net assimilation rate (Fu et al., 2023).

In addition, appropriate tillage practices and irrigation levels are crucial for regulating the photosynthetic source of maize populations (Cong et al., 2024). The present study found that the integration of no-tillage with medium irrigation in an intercropping system significantly increased leaf area index, leaf area duration, and net assimilation rate from silking to milk stages. This is consistent with previous studies reporting that under limited irrigation, no-tillage helps maintain a higher photosynthetic source level during the mid- and late growth stages, thereby delaying leaf senescence and enhancing photosynthetic efficiency (Wang et al., 2025). On the one hand, under medium irrigation, no-tillage intercropping promotes root growth, enabling more efficient use of limited soil water, improving water use efficiency, and stabilizing the internal leaf environment. This enhances leaf stress resistance, delays chlorophyll degradation and the decline in photosynthetically active leaf area, thereby maintaining greater leaf area index, leaf area duration, and net assimilation rate during the later growth stages and providing a solid basis for sustained photosynthesis (Niu et al., 2023). On the other hand, under limited irrigation, no-tillage combined with intercropping reduces soil mechanical damage and erosion, minimizes nutrient loss and water evaporation, and improves the coordination between maize roots, soil water, nutrients, and temperature (Li et al., 2025). This enhances nutrient and water uptake, thereby promoting leaf growth in the later stages, indirectly extending leaf greenness duration, and increasing leaf area index, leaf area duration, and net assimilation rate (Adil et al., 2024; Zhang et al., 2022). Thus, applying no-tillage to intercropping systems under medium irrigation can promote root development, delay leaf senescence, better coordinate photosynthetic source dynamics during growth, extend the grain-filling period, and expand post-silking photosynthetic area. This effectively enhances photosynthesis and sustains higher maize grain yield (Martínez-Goñi et al., 2023; Zhang et al., 2022).

Collectively, these results highlight that the integration of no-tillage and intercropping is effective in optimizing canopy structure and extending photosynthetic duration, providing a stronger source capacity to support grain filling under deficit irrigation regimes.

### 4.3 Water use efficiency and resource complementarity in the intercropping system

Water-use efficiency is a key indicator of sustainable crop production in arid and semi-arid regions. In this study, no-tillage intercropping under medium irrigation (NTIMI2) achieved the highest water use efficiency, outperforming both conventional tillage and monocropping treatments. This improvement can be attributed to the combined effects of enhanced canopy photosynthesis and moderated water consumption, which allowed plants to generate more biomass and grain yield per unit of water used. Similar observations have been reported in oasis irrigation systems, where optimized irrigation and tillage practices improved water-use efficiency without compromising yield (Awe et al., 2021; Martínez-Goñi et al., 2023).

The advantage of intercropping systems lies in their complementary use of belowground and aboveground resources. Legume species, such as pea in our experiment, enhance soil nitrogen availability through biological fixation, reduce interspecific competition, and improve root distribution patterns, thereby facilitating maize access to soil moisture and nutrients. Previous studies demonstrated that cereal–legume intercropping increases root length density in deeper soil layers, improving water uptake under limited irrigation (Li et al., 2020; Mao et al., 2012; Raza et al., 2022). Additionally, no-tillage practices reduce soil disturbance, enhance soil organic matter, and improve soil water-holding capacity, which further supports efficient water use.

Together, these results suggest that integrating no-tillage with maize–pea intercropping and medium irrigation provides a practical pathway to balance high yield with high water use efficiency. Such strategies can mitigate the trade-off between productivity and resource conservation, which is critical for maintaining food security in water-scarce environments.

## 5 Conclusions

This study demonstrated that the integration of no-tillage, intercropping, and medium irrigation is an effective strategy to stabilize grain yield and improve resource-use efficiency in maize production under water-limited conditions. Compared with conventional tillage and monocropping, no-tillage intercropping significantly increased grain yield by enhancing ear number and thousand-kernel weight, while maintaining comparable productivity under medium irrigation to that of high irrigation. At the physiological level, no-tillage intercropping improved canopy traits such as leaf area index, leaf area duration, crop growth rate, and net assimilation rate during the critical silking–filling stages, thereby sustaining canopy photosynthetic activity and extending the functional duration of green leaves. Enhanced water use efficiency was achieved through the synergistic effects of reduced soil water loss, improved soil–plant water relations, and resource complementarity between maize and pea. Moreover, the upregulation of photosynthetic enzyme activities and related gene expression during grain filling provided additional biochemical support for assimilate production and partitioning. Collectively, our findings highlight that no-tillage intercropping combined with medium irrigation not only secures high maize yield but also promotes water-use efficiency, offering a promising agronomic approach for sustainable intensification in arid irrigated regions. Future studies should validate these results across multiple years and environments, and integrate remote sensing and modeling tools to facilitate large-scale applications.

## Data Availability

The raw data supporting the conclusions of this article will be made available by the authors, without undue reservation.
