# Supplementary material for: No-tillage combined with deficit irrigation improves canopy photosynthesis and water use efficiency to stabilize yield in intercropped maize
Source: Front Plant Sci. 2026 Jan 27;16:1712975. doi: 10.3389/fpls.2025.1712975 (PMC12887593; doi:10.3389/fpls.2025.1712975)
Supplement: Supplementary file 1 [file DataSheet1.doc]

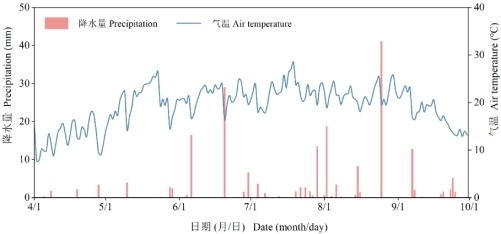


**Fig. S1** Precipitation and temperature changes in the study area in 2024

**Table. S1** Experimental design

| **Tillage initiatives** | **Planting pattern** | **Irrigation amounts (m^3^ hm^–2^)** | | |
| --- | --- | --- | --- | --- |
|  |  | **I1 (3900)** | **I2 (4650)** | **I3 (5400)** |
| No-tillage (NT) | IM | NTIMI1 | NTIMI2 | NTIMI3 |
|  | SM | NTSMI1 | NTSMI2 | NTSMI3 |
| Conventional tillage (CT) | IM | CTIMI1 | CTIMI2 | CTIMI3 |
|  | SM | CTSMI1 | CTSMI2 | CTSMI3 |

**Table. S2** Irrigation quotas for maize at different growth stages under varying irrigation amounts

| **Irrigation amounts** | **Irrigating water quota (m^3^ hm^–2^)** | | | | | |
| --- | --- | --- | --- | --- | --- | --- |
|  | **Seeding** | **Jointing** | **Big flare** | **Silking** | **Filling** | **Irrigation quota** |
| I1 | 900 | 900 | 900 | 900 | 900 | 4500 |
| I2 | 900 | 1050 | 1050 | 1050 | 900 | 4950 |
| I3 | 900 | 1200 | 1200 | 1200 | 900 | 5400 |

**Table. S3** The primer sequences for gene expression

| **Gene Symbol** | **Forward primer(5->3)** | **Reverse primer(5->3)** | **Product length**  **(bp)** | **Tm**  **(℃)** |
| --- | --- | --- | --- | --- |
| LOC542374 *nadp-mdh* | TCGGACAAGACCAACCAAT | TCAGCAGTGGATACAGCGA | 108 | 60 |
| LOC542233 *nadp-me* | GTCTTGCTTTCACAGAGGAG | GAGTTCCTGAGATAGTACTGCC | 80 | 60 |
| Maize *actin* | AGATCATGTTCGAGACCTTCA | ACTGGCATACAGAGAGAGCA | 74 | 60 |
